# A Systematic Review to Explore a Neuropsychological Profile that Predates Anorexia Nervosa

**DOI:** 10.1093/arclin/acae072

**Published:** 2024-09-07

**Authors:** Rachel Noon, Tayeem Pathan

**Affiliations:** Division of Medicine, Eating Disorders and Clinical Nutrition, UCL, London, UK; Division of Medicine, MRCPsych Honorary Clinical Lecturer, Consultant Psychiatrist in Eating Disorders, Surrey and Borders Partnership NHS Foundation Trust, UCL, London, UK

**Keywords:** Anorexia nervosa, Endophenotype, Neuropsychology, Systematic review

## Abstract

**Objective:**

Research demonstrates reduced cognitive flexibility and weak central coherence during acute illness and following recovery from anorexia nervosa (AN). This systematic review investigated if these impairments are present in first-degree relatives of individuals with AN, representing a possible neuropsychological risk profile.

**Methods:**

A systematic review of electronic databases was conducted following the Preferred Reporting Items for Systematic Reviews and Meta-Analyses guidelines. The search ended on July 14, 2023. Established search terms and inclusion criteria identified relevant research. Risk of bias was assessed using the Critical Appraisal Skills Program. The review was registered with Prospero international prospective register of systematic reviews (No. CRD42023401268). Study selection, descriptive data, critical appraisal, and risk of bias are presented in tables and figures.

**Results:**

The search yielded 10 studies. The included studies conducted neuropsychological assessments of discordant AN relatives and lifetime longitudinal study participants. Most studies found cognitive flexibility and central coherence to be significantly reduced in participants with AN and their relatives compared with controls. One study found decision making to be significantly impaired in AN participants and relatives. Effect sizes were moderate to large.

**Discussion:**

Reduced cognitive flexibility and weak central coherence appear to be endophenotypes of AN. Further research is required with relatives concordant for AN to establish whether these biomarkers co-segregate with AN within families. These findings suggest a possibility of developing screeners to identify individuals at risk of AN allowing for early intervention.

## INTRODUCTION

### Anorexia Nervosa: Classification and Update on Epidemiology

Anorexia nervosa (AN) is a disorder with high mortality. Research has demonstrated the standardized mortality rate of AN to be higher than other eating disorders (EDs; Arcelus et al., 2011) and all other unrelated psychiatric conditions (Walker et al., 2015). The standardized mortality rate of AN is 5.86 compared with bulimia nervosa and other specified feeding or EDs that have an average standardized mortality rate of 1.92 (Arcelus et al., 2011) and other psychiatric conditions with an average standardized mortality rate of 2.22 (Walker et al., 2015).

The diagnostic criteria for AN in the Diagnostic and Statistical Manual of Mental Disorders–Fifth Edition (DSM-5) include restricted eating resulting in low body weight, extreme fear of weight gain and body image disturbance (American Psychiatric Association, 2013). There are subtypes within this diagnosis: AN restricting type (without bingeing and/or purging) and AN binge-eating/purging type (with bingeing and/or purging). The DSM-5 also includes severity thresholds based on body mass index (BMI): mild (≥17 kg/m^2^), moderate (16–16.99 kg/m^2^), severe (15–15.99 kg/m^2^), and extreme (<15 kg/m^2^; American Psychiatric Association, 2013).

Contemporary research suggests the etiology of AN follows the biopsychosocial model (Canals and Arija Val, 2022). There is a background of research from before the turn of the 21st century that indicated the presence of genetic links in AN (Holland et al., 1984; Lilenfeld et al., 1998; Strober et al., 2000). Early family studies (Lilenfeld et al., 1998; Strober et al., 2000) identified increased incidence of AN diagnostic criteria in first-degree family members of individuals with AN compared with healthy controls. Early twin studies (Holland et al., 1984) demonstrated greater concordance of AN among monozygotic (MZ) compared with dizygotic (DZ) twins. However, these genetic studies had small sample sizes and were conducted over 20 years ago, therefore the findings may lack internal and temporal validity. Furthermore, the inclusion criteria were different to what they would be if this research was repeated today (Zipfel et al., 2015). These studies were conducted while the DSM third and fourth editions were in use, which had different diagnostic criteria for AN than the current DSM-5 (such as the presence of amenorrhea, which is no longer required for diagnosis in the DSM-5; Zipfel et al., 2015). Therefore, the diagnostic criteria from these earlier editions may not have been wide enough to capture all individuals with AN. Research suggests prevalence rates for AN have increased from ~1.2% and 0.29% in the early 2000s (Bulik et al., 2006) to 4% and 0.3% in 2021 (van Eeden et al., 2021) for women and men, respectively. This could be attributed to the changes to the diagnostic criteria between these time periods (Zipfel et al., 2015). AN is diagnosed more frequently in women compared with men (van Eeden et al., 2021). These current figures were established in a review of novel research from psychiatric settings and the general population in Westernized countries. There is a definitive lack of research into prevalence rates of AN in non-Western countries. The review by van Eeden et al. (2021) identified two Taiwanese studies which reported much lower overall prevalence rates (~0.0013%). This may be associated with different cultural idyllic body images (Swami and Tovée, 2005) or Westernized diagnostic methods may be insensitive to cultural differences (Simpson, 2002). Currently due to the limited research, it is unclear.

### Neuropsychological Profile in Anorexia Nervosa

Research has examined various neuropsychological domains in AN. These domains primarily include general intelligence, cognitive flexibility, and central coherence.

#### General intelligence

The Wechsler Intelligence Scale for Children (WISC; Wechsler, 2014) and Wechsler Adult Intelligence Scale (WAIS; Wechsler, 2008) appear to be reliable measures of general intelligence (Nelson et al., 2013; Canivez et al., 2019). Research suggests that starvation negatively affects some cognitive functions (Lozano-Serra et al., 2014; Pender et al., 2014); however general intelligence does not appear to be affected. On the contrary, research has identified that on average, even individuals with acute AN appear to have higher intelligence quotients (IQs) than the general population (Schilder et al., 2017). It has been suggested that increased IQ may be a contributing factor to its development (Schilder et al., 2017).

#### Cognitive flexibility

The Wisconsin Card Sorting Test (WCST; Berg, 1948) has been found to have adequate ecological validity (Chiu et al., 2018) and content validity (Faustino et al., 2021). Research using the WCST has found that individuals who are acutely ill with AN perform more poorly on measures of cognitive flexibility and higher on measures of perfectionism than healthy controls (Buzzichelli et al., 2018). Research suggests that starvation may reduce set-shifting abilities (Pender et al., 2014). This research investigated the effect of short-term fasting on cognitive abilities in healthy participants using a measure similar to the WCST. However, further research suggests that impaired set-shifting may be associated with the etiology of AN, because even individuals of healthy weight in long-term recovery have been found to have reduced set-shifting abilities on the WCST (Tenconi et al., 2010). However, the use of the measure that was similar to, but not, the WCST in the research by Pender et al*.* may make the findings less reliable. Generally, findings of the effect of starvation on cognition are mixed (Benau et al., 2014). Furthermore, the effects of short-term fasts on healthy individuals may not be generalizable to acutely ill individuals with AN, as there are many metabolic adaptations that occur during longer term starvation, such as reduced metabolic rate and increased levels of cortisol to preserve energy (Wassif and Ross, 2013). These adaptions could affect cognitive function (Nikendei et al., 2011) meaning that individuals with AN may perform differently to healthy participants in a fasted state.

#### Central coherence

The Navon task (Navon, 1977) has been validated as a measure of processing styles (Staudinger et al., 2011). Weak central coherence, where holistic processing is diminished, was first investigated in individuals with autism spectrum disorder (ASD; Frith and Happé, 1994), but also appears to be a characteristic of AN both during acute illness and following weight restoration (Danner et al*.*, 2012), therefore suggesting that it too may be involved in the etiology of AN. However, multiple studies have demonstrated that unlike in ASD, individuals with AN do not appear to prefer localized processing, but instead exhibit global processing deficits (Tenconi et al., 2010) and greater local interference (Weinbach et al., 2017).

### Existing Research and its Shortcomings

Reviews have been conducted collating the existing research into whether AN has lasting neuropsychological impact (Steinglass and Glasofer, 2011; Solano-Pinto et al., 2018; Fuglset, 2019). The review by Steinglass and Glasofer (2011) concluded that deficits in reaction-time, memory, and visuospatial abilities during acute illness with AN appeared to return to typical levels following weight-restoration. This review highlighted set-shifting as a neuropsychological ability that continues to be impaired in individuals with AN who are weight restored (Steinglass and Glasofer, 2011), which is a common finding from the research (Tchanturia et al., 2004; Tenconi et al., 2010; Danner et al., 2012). The most recent review was conducted in 2019, which concluded that there appear to be lasting neuropsychological deficits, particularly in set-shifting, following recovery from AN (Fuglset, 2019). However, these reviews often include research with methodological issues. Research often defines recovered individuals as those with a BMI of >18.5 kg/m^2^ and resumption of a menstrual cycle for at least 12 months (e.g., Danner et al., 2012; Tchanturia et al., 2012). However, despite their weight restoration, these individuals may not be fully recovered, and the findings could still reflect the cognitions of acute AN (Couturier and Lock, 2006).

Fewer reviews have investigated whether neuropsychological deficits predate the development of AN. A review by Lena et al*.* (2004) and the more recent review by Fuglset (2019) concluded that this may be the case. However, these conclusions were based on studies of individuals recovered from AN. Neuropsychological deficits following recovery do not demonstrate that these deficits existed before the development of AN as they may have resulted from the illness. Therefore, the aim of this review was to examine the current available research investigating neuropsychological deficits in individuals prior to the development of AN to establish if there are apparent neurocognitive risk factors associated with the later development of AN. If there is a particular neuropsychological profile that is present before the development of AN this would have implications for clinical practice. The identification of such a profile could allow for early screening and preventative measures for individuals likely to be at risk of developing AN, potentially allowing for prevention or very early help, likely improving outcomes for patients (Steinhausen, 2009). The hypothesis of the current review predicts that set-shifting and central coherence are impaired in individuals prior to the development of AN. If this is the case, perhaps there are interventions that could be utilized pre-emptively to improve these cognitive abilities before individuals develop AN. For example, cognitive remediation therapy which has been found to be effective at improving set-shifting and central coherence in individuals with AN (Rhind et al., 2022).

## MATERIALS AND METHODS

### Literature Search

This systematic review followed the protocol for transparent reviews detailed in the Preferred Reporting Items for Systematic Reviews and Meta-Analyses (PRISMA) guidelines (Page et al., 2021). The searches were conducted in stages. The following electronic databases were searched for relevant published research: PubMed, Google Scholar, Cochrane CENTRAL, PsychINFO. To search for gray literature the database PsychEXTRA was used. The International Clinical Trials Registry (ICTRP) was used to search for unpublished relevant research. Once all electronic databases were exhausted, a manual search of the reference lists of included studies was conducted. The search was conducted from June 27 to July 14, 2023. Search terms included (prospective cohort study OR longitudinal study) AND (anorexia nervosa) AND (neuropsychology OR executive function OR set-shifting OR cognitive flexibility OR central coherence OR inhibition response OR inhibitory control OR working memory OR attention OR planning OR visuospatial abilities OR general intelligence OR IQ). For databases where very large numbers of studies were yielded based on the search criteria (Google Scholar, PsychInfo, and ICTRP), the first 20 pages of results were screened.

### Selection Criteria

See Table 1.

**Table 1:** Inclusion and exclusion criteria

Criteria	Include	Exclude
1. Participants	Participants with a diagnosis of AN based on any versions of the DSM Healthy control participants	Participants with related diagnosed comorbidities (i.e., articles including participants diagnosed with ASD or OCD) No participants with AN (i.e., articles including only participants with other EDs – BN, BED etc.) Participants with anorexia as a result of comorbid physical illness rather than AN
2. Measures	Neuropsychological measures	Lack of neuropsychological measures Measures of disordered eating behaviors rather than AN diagnosis based on DSM diagnostic criteria
3. Design	Prospective cohort design prior to the onset of AN Empirical research design	Reviews or single case study designs
4. Language	Published in or translated to English	

Note. AN = anorexia nervosa, DSM - diagnostic and statistical manual of mental disorders, ASD = autism spectrum disorder, OCD = obsessive compulsive disorder, EDs = eating disorders, BN = bulimia nervosa, BED = binge eating disorder.

### Screening for Eligibility

All papers were initially screened for relevance through their titles and abstracts. Seemingly relevant titles/abstracts were selected for further screening for inclusion/exclusion criteria through the full article content. There was a further independent screening conducted by the second author to establish agreement over selected articles and control for selection bias.

### Data Extraction

Data were extracted using a standardized extraction form based on the Cochrane standardized extraction form (Higgins et al., 2022; Appendix A). This was piloted prior to the search being conducted to ensure that all important data was captured. This collected study characteristics, participant characteristics, the study research methods and outcomes. The Critical Appraisal Skills Program checklists for case control (2018a) and cohort studies (2018b) were used to assess the quality of evidence and risk of bias in the studies included in the review.

### Registration

This systematic review was registered with Prospero international prospective register of systematic reviews. Registration number: CRD42023401268.

## RESULTS

### Study Selection

**Fig. 1:**
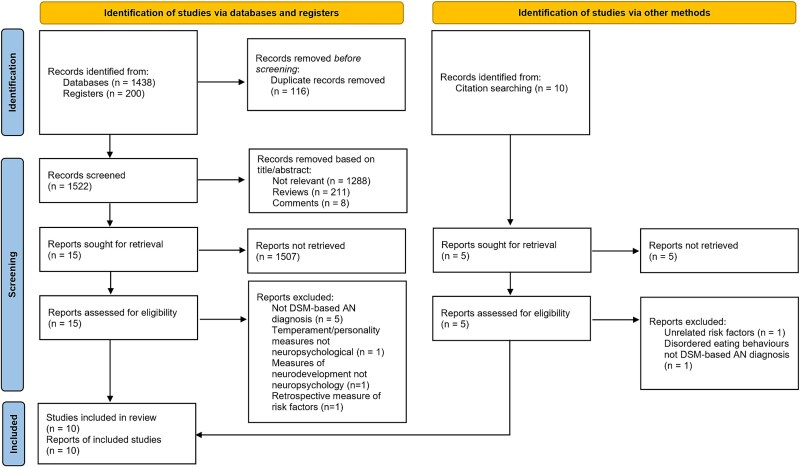
PRISMA 2020 flow diagram of study selection.

Figure 1 presents the PRISMA flow diagram (Page et al., 2021) recording the process for the selection of studies to be included in the review. An initial 1,438 reports were identified through electronic database searches and an additional 200 were identified through searching registers. After removing duplicate records which amounted to 116 reports, 1,522 studies were available for screening using the inclusion and exclusion criteria. Preliminary screening based on the titles and abstracts of these reports excluded a total 1,507. The majority of studies were excluded because they were not relevant to the research question. This left 15 reports for retrieval and further screening on the whole articles. Five of these reports were excluded for not using DSM diagnostic criteria; two were excluded for not using neuropsychological measures and one was excluded due to being a retrospective rather than prospective measure of risk factors. The manual citation search yielded an initial 10 relevant articles for screening. Of these, following screening of the abstracts, five were excluded because they were not relevant to the research questions. For the remaining five reports the whole articles were screened. One article was excluded because it investigated risk factors unrelated to neuropsychology and one was excluded because it included participants with disordered eating behaviors rather than DSM diagnosed AN. Overall, this left 10 reports to be included in the review.

### Findings

Key methodological details and findings from the included studies are depicted in Table 2. Of the total 10 included studies, one was a twin study researching twin pairs where one twin was concordant for AN and the other was discordant, compared with control twin pairs. One study investigated individuals concordant for AN and their unaffected first-degree relatives, who were a combination of biological mothers and sisters, compared with control sister and mother-daughter pairs. One study investigated mothers of children with AN, compared with control mothers. A further four studies investigated sister pairs where one was concordant for AN and the other was discordant. Two investigated children born to mothers with AN. The final study was a lifetime longitudinal study, where neuropsychological measures taken in childhood were analysed for participants who later developed AN.

**Table 2:** Neuropsychological research findings in first-degree relatives of individuals with anorexia nervosa

**Authors**	**Measurements**	**Participants (n:mean age)** **(m = months)**	**Key findings**	**Effect size (Cohens *d*)**	**Author conclusions**
Kanakam et al., 2013	WCST, Brixton task (for set-shifting) ROCFT, GEFT (for central coherence) NART (for estimated IQ functioning)	AN (26:_) [MZ-ED (41:31) DZ-ED (12:35)] Non-AN cotwin (12:_) [MZ-H (11:54) DZ-H (8:52)] C twins (42:45)	AN (*p* = .04) and UN twins (*p* = .28) made more errors on WCST and Brixton task (AN: *p* = .77; UN cotwin: *p* = .57) than C AN scored higher on ROCF (*p* = .2) and UN cotwins scored lower (*p* = .10) than C AN (*p* = .32) and UN cotwins (*p* = .77) had reduced time on GEFT	WCST: *d* = 0.6 for AN, *d* = 0.4 for UN cotwins Brixton: *d* = 0.1 for AN, *d* = 0.2 for UN cotwin ROCF: *d* = 0.3 for AN, *d* = −0.6 for UN cotwin GEFT: *d* = −0.3 for AN, *d* = −0.1 for UN cotwin	Central coherence and cognitive flexibility appear to be endophenotypes associated with eating disorders
Galimberti et al., 2013	IGT (for decision making) ToH (for planning) WCST (for set-shifting)	AN (29:24.1) Unaffected AN relative (29:43.8) C (29:28.6) C relative (29:43.3)	AN and relatives performed more poorly than C and relatives on IGT(*p* < .05), ToH (*p* > .05) and WCST (*p* < .05)	IGT: *d* = 0.8 for AN, *d* = 1.3 for UN relatives ToH: *d* = 0.7 for AN, *d* = 0.8 for UN relatives WCST perseverative errors: *d* = 0.7 for AN, *d* = 3.4 for UN relatives WCST total errors: *d* = 0.7 for AN, *d* = 1.1 for UN relatives	Findings suggest a shared dysfunctional executive profile in women with AN and their unaffected relatives, characterized by impaired decision making and cognitive flexibility
Lang et al., 2016	WASI (for IQ) WCST (for set-shifting) ROCFT and FPT (for central coherence)	UN AN mothers (21:48.9) C mothers (20:47.8)	UN AN mothers made more errors on WCST (*p* < .01) and scored lower on central coherence measures (*p* < .05)	WCST: *d* = 0.81 FPT: *d* = 0.88 ROCFT CCI: *d* = 0.71	Findings support that inefficient cognitive processing may be a familial trait in AN
Holliday et al., 2005	Haptic illusion task (for perception and set-shifting) Brixton task, CatBat task (for set-shifting) TMT (for visual attention and set-shifting) NART, WAIS (for IQ)	AN (47:26.3) UN AN sisters (47:27.6) C (47:26.5)	No significant differences in IQ (*p* = .25) AN and UN sisters scored more poorly on Haptic illusion task (*p* < .001). AN and UN sisters did not significantly differ on any measures of cognitive flexibility (*p* > .01)	Haptic illusion: *d* = 0.9 for AN *d* = 0.7 for UN relatives CatBat: *d* = 0.62 for AN, *d* = 0.64 for UN relatives Brixton: *d* = 0.3 for AN, *d* = 0.5 for UN relatives	AN sisters and UN sisters had more impairments in set-shifting than C, suggesting they are trait characteristics
Roberts, 2009	WCST, Haptic illusion, CatBat task, Brixton and TMT (for set-shifting) Block design task, EFT, ROCFT and GEFT (for central coherence)	AN (30:24.1) AN UN sisters (30:24.2) C (88:28.4)	AN and UN sisters made significantly more errors on the WCST than C (*p* < .05) AN and UN sisters had significantly faster reaction times on the GEFT (*p* < .05) and lower CCI (*p* < .01) than C	For AN and UN relatives: WCST *d* = 0.49 Brixton *d* = 0.08 CatBat *d* = 0.16 GEFT *d* = 0.37 ROCF CCI *d* = 1.14	Sisters of women with diagnoses of ED demonstrated reduced cognitive flexibility and weak central coherence compared to C

(*Continued*)

**Table 2:** Continued

**Authors**	**Measurements**	**Participants (n:mean age)** **(m = months)**	**Key findings**	**Effect size (Cohens *d*)**	**Author conclusions**
Tenconi et al., 2010	ROFT, Block Design and Object Assembly subtest of the WAIS, Overlapping Figures Test (for central coherence) WCST and TMT (for set-shifting)	AN (153:26.2) UN AN sisters (56:27.5) C (120:27.4)	AN and UN sisters did not differ significantly on any of the neuropsychological tasks (*p* > .05) except for the ROFT Central Coherence Index (*p* < .05) and Copy Order Index (*p* < .005). AN and UN sisters performed poorer on WCST (*p* < .05) and TMT B (*p* < .005)	For AN: WCST perseverative errors: *d* = 0.38 TMT B: *d* = 0.59 ROCF CCI: *d* = 0.47	Cognitive flexibility and central coherence appear to be promising endophenotypes for AN
Kothari, 2013	WISC-III (for general intelligence) TEA-Ch (for attention) Counting Span Task (for working memory) Stop-signal Task (for inhibition control) Reaction time (for focused and sustained attention)	Tested at 8^1/2^, 10^1/2^ and 13^1/2^: Children with AN mothers (127:103.8 m), (96:127.8 m), (82:150 m) Unexposed (670:103.8 m), (643:127.8 m), (_:150 m)	Children of women with restrictive phenotypes demonstrated significantly higher IQ scores than unexposed children or children to mothers with BN phenotypes (*p* = .03) No differences found between children at risk of restriction and unexposed children on working memory, attention, reaction times or inhibition (*p* > .05)	_	Superior intellectual functioning may be present in high-risk subjects
Roberts et al., 2013	GEFT and ROCFT (for central coherence)	AN-R (35:23.7) AN-BP (33:25.6) AN recovered (30:32.1) AN UN sisters (30:24.2) C (88:28.4)	AN-R and recovered were significantly faster on GEFT than C (*p* = .03) AN-R/BP had lower central coherence index than C (*p* = .004) No differences between UN sisters and AN (*p* > .05) 45% of AN ppts and their sisters demonstrated weak central coherence	UN sisters: GEFT: *d* = 0.35 ROCF CCI: *d* = 0.92	Attention to detail is a strong endophenotype for AN
Pappaianni et al., 2023	WASI-II (for general intelligence) The attention switching task (for cognitive flexibility) Rapid visual information processing (for sustained attention) Spatial Working Memory task (for working memory)	UN daughters of AN mothers (14:12) C (18:12.3)	Maternal risk for AN scored higher on the cognitive flexibility task than C (*p* < .05; indicating slower switching times) UN AN daughters were not significantly different to C on working memory or attention tasks (*p* > .05) There were no significant differences for IQ (*p* = .93)	_	Impaired cognitive flexibility is a potential endophenotype for AN.
Schaumberg et al., 2020	TEA-Ch (for attention) Freedom from distractibility index from WISC-III, Counting Span Task (for working memory) Stop-signal Task, Opposite worlds task from TEA-Ch (for inhibition control)	Neuropsychological tests at ages 8 and 10, AN assessment at ages 14, 16 and 18 Total sample = 2119% of AN boys at 14 = 1.5%, 16 = 1.5%, 18 = 1% % of AN girls at 14 = 3.7%, 16 = 2.5%, 18 = 1.8%	No significant predictors for AN (*p* > .05)—but were unable to look at set-shifting or central coherence	Working memory odds ratio = 0.99 (*p* > .05) for AN Inhibition odds ratio = 1.00 (*p* > .05)	The current findings suggest that neuropsychological alterations may not be particularly useful to flag eating disorder risk

Note. AN = Anorexia Nervosa, UN = unaffected, AN-R = Anorexia Nervosa restrictive subtype, AN-BP = Anorexia Nervosa binge/purge subtype, MZ-ED = Monozygotic eating disorder probands, DZ-ED = Dizygotic eating disorder probands, MZ-H = Monozygotic non-eating disorder cotwin, DZ-H = Dizygotic non-eating disorder cotwin, C = control, WCST = Wisconsin Card Sorting Test, ROCF = The Rey-Osterrieth Complex Figure test, FPT = Fragmented Pictures Task, CCI = central coherence index, GEFT = Group Embedded Figures Test, IGT = the Iowa Gambling Task, ToH = the Tower of Hanoi, WASI = The Wechsler Abbreviated Scale of Intelligence, WAIS = Wechsler Adult Intelligence Scale, TMT = Trail Making Test, EFT = Embedded Figures Test, TEA-Ch = Test of Everyday Attention for Children, WISC = Wechsler Intelligence Scale for Children, NART = National Adult Reading Test.

From the paper by Roberts (2009) only study four was included in this review because the other studies were not relevant to the research question. On this same basis, only study two from the Kothari (2013) paper was included. One other study in this paper was relevant but did not use DSM diagnostic criteria for the inclusion of AN participants. Some of the included studies used additional measures but these were not recorded as they were not relevant to the research question. Furthermore, some studies also included participants with BN but these data were not recorded as they were not relevant to the review research question.

Based on definitions from previous research (Roberts et al., 2009), the Cohen’s *d* effect sizes may be considered as small (<0.4), moderate (0.4–0.75), large (0.75–1.10), and very large (>1.10).

#### Cognitive flexibility

Of the 10 included studies, seven investigated cognitive flexibility as a measure of neuropsychology. Of these, six found cognitive flexibility to be significantly impaired in first-degree relatives of individuals with AN. Kanakam et al*.* (2013) found that the twins meeting diagnostic criteria for AN and their non-AN cotwins made more errors on the WCST and Brixton task than controls, indicating reduced cognitive flexibility. However, these findings only reached significance for the individuals with AN with small to moderate effect sizes. The unaffected cotwins did not score significantly differently to control participants. Despite this, cognitive flexibility abilities were more similar in the MZ twins (WCST: *r* = .26, *p* = .53; Brixton task: *r* = .22, *p* = .13) compared to the DZ twins (WCST: *r* = −.39, *p* = .88; Brixton task: *r* = −..04, *p* = .55), indicating heritability. Galimberti et al*.* (2013), Lang et al*.* (2016) and Roberts (2009) found that participants with AN and their unaffected relatives made significantly more errors on the WCST than controls with large-moderate effect sizes. They found that there were no significant differences between the scores of individuals with AN and their unaffected relatives. Holliday et al*.* (2005) found that participants meeting criteria for AN and their unaffected sisters had significantly longer response times on the CatBat and Haptic Illusion task than controls with small to moderate effect sizes, indicating poorer cognitive flexibility. Participants with AN and their unaffected sisters did not significantly differ in scores from any of the measures of cognitive flexibility. Roberts (2009) also found no significant differences between the unaffected sisters and controls for the TMT, Haptic Illusion, or Brixton task. Tenconi et al*.* (2010) found that AN participants and their unaffected sisters performed more poorly on the measures of cognitive flexibility than control participants with small to moderate effect sizes. Pappaianni et al*.* (2023) found that individuals who were at maternal risk of AN performed more poorly in the cognitive flexibility task compared to control participants. However, it is important to note that there was significant variability found between the studies, therefore conclusions remain tentative.

#### Central coherence

Five of the included studies used central coherence as a measure of neuropsychological abilities. Of these, four studies found weak central coherence in first-degree relatives of individuals with AN. Roberts (2009) and Roberts et al*.* (2013) found that participants with AN and their unaffected sisters had significantly faster reaction times on the GEFT, indicating a preference for localized processing. Roberts (2009) found both groups also remembered significantly less at the 20 min recall than control participants, demonstrating reduced global processing. Roberts (2009) and Lang et al*.* (2016) found that first-degree relatives of individuals with AN scored significantly lower CCI on the ROCF than controls. Tenconi and coworkers (2010) produced mixed results for central coherence. AN participants and their unaffected sisters had poorer performance on the Overlapping Figures Test, Block Design task, and Object Assembly than controls. However, although performance of AN participants was reduced on the ROCFT and copy order index, this was not the case for the unaffected sisters. In contrast, Kanakam et al*.* (2013) found that participants with AN scored higher on the CCI, indicating enhanced central coherence. However, this finding did not reach significance. Central coherence scores were more similar in MZ twins (ROCF: *r* = .44, *p* = .01; GEFT: *r* = .58, *p* < .001) than DZ twins (ROCF: *r* = −.37, *p* = .87; GEFT: *r* = .18, *p* = .48), indicating some heritability of the trait. Similarly to with cognitive flexibility, there was significant variability in the findings between studies and there were few studies to draw from therefore conclusions remain tentative.

#### General intelligence

Five of the included studies investigated general intelligence as a measure of neuropsychological function. The majority of these studies found no significant differences between IQ scores in first-degree relatives of individuals with AN compared to controls (Holliday et al., 2005; Kanakam et al., 2013; Lang et al., 2016; Pappaianni et al., 2023). Kothari (2013) found that children of women with restrictive ED phenotypes had significantly higher IQ scores than unexposed children or children to mothers with binge/purge phenotypes.

#### Other neuropsychological abilities

**Fig. 2:**
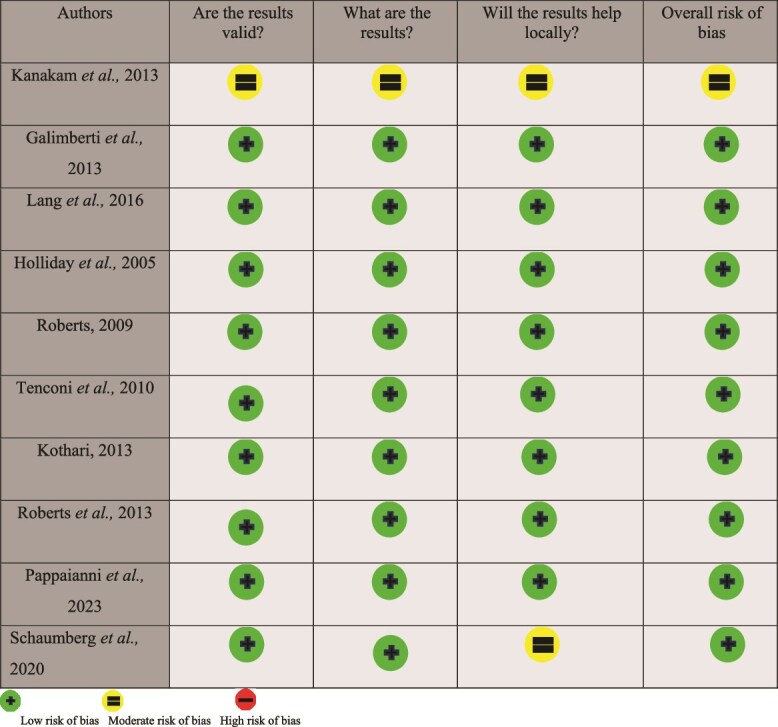
Risk of bias of included studies.

Galimberti et al*.* (2013) found decision making abilities to be reduced in first-degree relatives of individuals with AN with a large effect size. Participants with AN and their relatives scored significantly lower on the IGT than controls. Kothari (2013) found no differences between children born to mothers with restricting phenotype EDs and unexposed children on working memory, attention, reaction times or inhibition. Pappaianni et al*.* (2023) and Schaumberg et al*.* (2020) found no significant differences between the performance of first-degree relatives of individuals with AN and controls on working memory or attention. Schaumberg et al*.* (2020) also found no significant differences for inhibition control (Fig. 2).

## DISCUSSION

The aim of this systematic review was to provide an overview of the literature investigating the neuropsychological profile of unaffected first-degree relatives of individuals with AN to attempt to investigate whether deficits predate AN rather than result from the illness. The review examined a total of 10 studies. As hypothesized, of the studies that investigated cognitive flexibility and central coherence, the majority found these neuropsychological functions to be impaired in some first-degree relatives of individuals with AN. As for other neuropsychological abilities, the evidence base is less consistent. Most studies measuring general intelligence found no differences between controls and first-degree relatives of AN sufferers. Only one study investigated decision making abilities and found these to be impaired in some first-degree relatives of individuals with AN. The studies that investigated working memory, attention, inhibition control and reaction times did not find any significant differences between some first-degree relatives of individuals with AN and controls. Prior to discussing the significance of the findings, it is important to note that AN appears to result from the accumulation of multiple factors including genetics, psychological state and environmental exposure (Canals and Arija Val, 2022). This paper was not attempting to establish cause of AN, but to investigate whether there is a neuropsychological profile which may increase vulnerability for the illness to allow for early identification of those who may be at risk. Research has identified that first-degree relatives of individuals with AN are 11.4 times more likely to develop AN themselves than people in the general population (Strober et al., 2000). As heritability is defined as the measure of variance in a population caused by variations in genes (Visscher et al., 2008), AN appears to be highly inheritable. The heritability of AN has been estimated at 70% (Gorwood et al., 2003), suggesting that genetics explain a large proportion of the variance between individuals who develop it and those who do not. This allows for the investigation of first-degree relatives to individuals with AN as people at high familial risk. Of course, there is no way of knowing whether all the first-degree relatives included in this research have a genetic vulnerability for AN. To know this would require genetic testing, which is not yet possible as a conclusive genetic profile of AN has yet to be identified (Paolacci et al., 2020), but it may be possible to identify an endophenotype (Gottesman and Gould, 2003). Therefore, this is important to consider while interpreting the findings.

### Neuropsychological Measures Used by the Selected Studies

The introduction of this review provided an overview to some of the more commonly used neuropsychological measures, but some of the studies included in the review used alternative measures.

The majority of the studies investigating cognitive flexibility used the WCST as a measure, which as previously discussed has been found to be a valid measure (Faustino et al., 2021). A number of studies also used the Brixton task to measure cognitive flexibility (e.g., Holliday *et al.,* 2005; Kanakam et al., 2013), which has also been found to be a valid measure (van den Berg et al., 2009). This task requires participants to predict patterns which are changed throughout the task, and like the WCST, is scored on the number of errors made (Kanakam et al., 2013). Two other frequently used tasks were the CatBat and Haptic Illusion tasks. The CatBat task involves participants reading a short story and filling in missing letters to complete words which need to change depending on the context (Roberts et al., 2007). This is also measured through perseverative errors. In the Haptic Illusion task participants are blindfolded while holding wooden balls of different sizes for a series of trials, after which they are given two balls of the same size and asked to judge their relative sizes. The experience of illusion where the balls are perceived to be different sizes indicates cognitive inflexibility (Roberts et al., 2007). These have both been found to be valid measures of cognitive flexibility (Tchanturia et al., 2004). However, both the WCST and Haptic Illusion task rely on sensory processing in addition to cognitive flexibility, therefore variances in this ability could have affected the findings.

Many of the studies measuring central coherence used the ROCFT. This task involves the participant copying a figure from a diagram, then reproducing it at a 3-min and 30-min recall, which is scored to produce an overall central coherence index (Zhang et al., 2021). It has been found to be a valid measure of central coherence (Poulton and Moffitt, 1995). However, it is important to note that the ROCFT also requires the use of visuospatial skills and working memory (Cardillo et al., 2022). Therefore, differing abilities on the ROCFT may reflect differences in these skills. Many studies also used the GEFT to measure central coherence. This measures the time taken to locate specific geometric shapes among unrelated shapes, with a shorter time demonstrating enhanced local processing (Kanakam et al., 2013). This has been validated as a measure of localized processing in research with EDs (Harrison et al., 2011). This measure also relies on visual processing therefore this may have had an impact on the findings. However, for both set-shifting and central coherence, the discrepancies in findings between some studies did not appear to be a result of the measures used, as the studies used many of the same validated measures.

The studies that measured general intelligence used a variety of measures. Some used the WISC and WAIS which have previously been discussed and have been found to be reliable measures (Canivez et al., 2019; Nelson et al., 2013). Included studies also used the WASI and NART, which have also been found to be valid and reliable measures of IQ (Crawford et al., 1989; Saklofske et al., 2000).

### Neuropsychological Profile of First-degree Relatives of Individuals With Anorexia Nervosa

#### Cognitive flexibility

The findings from the majority of the studies included in this review suggest that cognitive flexibility could be impaired prior to the development of AN. Most studies found cognitive flexibility to be significantly reduced in both individuals diagnosed with AN and their unaffected first-degree relatives compared to controls. This suggests that cognitive flexibility may be a heritable trait, which previous reviews have also suggested (Fuglset, 2019), and that impairments may increase vulnerability for developing AN. The twin study included in this review found more similarities between the cognitive flexibility of MZ twins than DZ twins suggesting potential heritability of the trait (Kanakam et al., 2013), as MZ twins share more genetic similarities than DZ twins (Huguet et al., 2017). It is important to note that the study by Kanakam et al. received the highest risk of bias score, therefore interpretations should be made very tentatively. However, all of the family studies included suggest some heritability of cognitive flexibility as abilities were shared between the first-degree relatives. Overall, most studies in this review found cognitive flexibility to be impaired in first-degree relatives of individuals with AN with moderate effect sizes, indicating that there were discernible differences between the scores of first-degree relatives of individuals with AN and controls. This goes some way to indicate that deficits in cognitive flexibility may predate AN and may contribute to the development of the disorder (Fuglset, 2019; Lena et al., 2004) by suggesting that the individuals who developed AN may have had the same neuropsychological profile as their first-degree relatives prior to the development of the disorder. Impaired cognitive flexibility may be associated with the development of behaviors observed in AN such as following rigid rules, routines, and rituals around food and eating (Friederich and Herzog, 2011). However, as previously mentioned, these findings remain tentative and more research is required.

#### Central coherence

When considering central coherence, it is important to note the difference between a preference for localized processing and impaired global processing. Impaired global processing is the inability to perceive things holistically, therefore causing weak central coherence. Whereas a preference for localized processing is having stronger abilities for local processing rather than global. Previous research with individuals with AN has found that during acute illness there appear to be global processing deficits (Tenconi et al., 2010) and greater local interference (Weinbach et al., 2017) causing weak central coherence. This is somewhat supported by the findings from this review. Roberts (2009) found that both individuals with AN and their first-degree relatives had impairments in global processing compared to controls. Lang et al*.* (2016) and Roberts et al*.* (2013) also found weak central coherence in individuals with AN and first-degree relatives. However, in contrast to the previous findings, Roberts (2009) found a preference for localized processing in both these groups instead. These studies found moderate to large effect sizes, indicating that central coherence was substantially reduced in first-degree relatives of individuals with AN compared to controls. Therefore, suggesting that weak central coherence may be a neuropsychological vulnerability factor for developing AN, presuming that this neuropsychological profile was shared by the individual with AN and their first-degree relative before they developed the disorder. Most studies found central coherence to be significantly impaired in first-degree relatives of individuals with AN compared to controls. This also suggests that central coherence may be a heritable trait, indicating that it is shared within families. The most recent previous systematic review (Fugslet, 2019) found mixed evidence for this. The current review also presents somewhat mixed findings. The twin study (Kanakam et al., 2013) found central coherence abilities to be more similar in MZ twins compared to DZ twins, indicating potential heritability. Roberts (2009) found central coherence to be reduced in individuals with AN and their unaffected relatives on both measures used (GEFT and ROCF). Whereas Tenconi et al*.* (2010) found that first-degree relatives of individuals with AN had poorer performance on some measures (Overlapping Figures Test, Block Design task and Object Assembly). Overall, from the included studies, both the findings from central coherence and cognitive flexibility remain tentative. Weak central coherence may be associated with the development of body image distortion in AN as individuals may focus on small details of their body perceived as imperfect (Gaudio and Quattrocchi, 2012).

#### General intelligence and other neuropsychological functions

This review did not find evidence of any other neuropsychological functions as vulnerability factors for the development of AN. Most studies found no significant differences in general intelligence between people with AN, their first-degree relatives or control participants. However, it is important to note that the studies investigated IQ across broad age groups, therefore potentially reducing the reliability of the findings. Further, Kanakam et al*.* (2013) used only the NART as its measure for IQ, which is merely a predictor of IQ functioning. Therefore, more research is required to establish whether IQ may be a contributing variable to the development of AN. From the studies that investigated working memory, inhibitory control and attention, there were no significant differences found in the abilities of first-degree relatives of individuals with AN or controls. Only one study investigated decision making abilities and found this to be significantly impaired in both participants with AN and their unaffected first-degree relatives (Galimberti et al., 2013). This finding also had a large effect size, indicating that there were substantial differences in the scores of relatives of AN participants and controls. Therefore, there may be scope for further research into decision making in the future.

#### Neuroimaging evidence?

When looking at a neuropsychological risk profile for AN, it is worth considering whether there is neuroanatomical evidence. Research has found gray matter reductions following weight restoration from AN, particularly in the anterior cingulate cortex (Martin Monzon et al., 2017; Mühlau et al., 2007). The anterior cingulate cortex appears to be associated with cognitive flexibility (Bissonette et al., 2013). As the current review suggests that impairments in cognitive flexibility may predate the development of AN, it may be that these differences in brain structures are also premorbid. One study included in this review investigated both neuropsychological profiles of individuals at risk of AN and resting brain activity through functional magnetic resonance imaging (fMRI; Pappaianni et al., 2023). These findings were not included in the review, because this was too wide for the research question at hand. To reiterate, Pappaianni et al*.* (2023) found that unaffected daughters of AN mothers had reduced cognitive flexibility compared with control participants. From the fMRI it was found that the daughters born to mothers with EDs demonstrated decreased functional connectivity in the default-mode network posterior areas and the medial superior frontal gyrus (Pappaianni et al., 2023). This may be associated with self-referential behaviors observed in AN (Northoff and Bermpohl, 2004), which research has demonstrated the frontal gyrus to be involved in (Morin and Hamper, 2012). They also found that participants at risk of EDs showed increased functional connectivity in the medial ventral visual compared with controls, in areas which have been found to be involved in face and object recognition (Pappaianni et al., 2023). It was suggested that these differences in processing may together contribute to distorted body image perception in EDs through changes to both visual and self-referential processing (Pappaianni et al., 2023). Increased visual processing in AN has been previously associated with weak central coherence (Li et al., 2015). This suggests that there is likely overlap between the neuropsychological and neuroanatomical risk factors for AN. Future researchers may wish to investigate this further to provide more robust evidence for this.

#### An endophenotype of anorexia nervosa?

As previously mentioned, these findings may contribute to the identification of an endophenotype of AN. According to Gottesman and Gould (2003) for a biomarker to be classified as an endophenotype it must be associated with illness in the population; be inheritable; be primarily state-independent; within families must co-segregate with the illness; and be found in nonaffected family members at a higher rate than the general population. Therefore, from the findings from this systematic review, cognitive inflexibility and weak central coherence meet most of these criteria, apart from the trait co-segregating with the illness within families, as concordant AN family members were not investigated. Therefore, future research may wish to investigate this further.

Further, research suggests that this endophenotype may be specific to AN rather than psychological conditions more generally. Donati et al*.* (2020) identified a different potential neuropsychological endophenotype for schizophrenia with impairments in attention and memory. There appear to be some similarities between an endophenotype for AN and obsessive compulsive disorder (OCD), but also some differences possibly differentiating the two disorders. Research indicates that OCD may be linked to a neuropsychological endophenotype characterized by impaired cognitive flexibility, inhibitory response, and memory (Rao et al., 2008).

**Table 3:** Strengths and limitations of included studies

**Authors**	**Strengths**	**Limitations**
Kanakam et al., 2013	Twin study allowing for analysis of heritability of traits Use of valid neuropsychological measures Unaffected cotwins and controls screened for ED Controls with family history of psychiatric illness excluded	Small sample size, which may increase the likelihood of type II errors Only female participants Volunteer sampling may reduce generalizability No demographic data provided for participants with AN
Galimberti et al., 2013	Use of valid neuropsychological measures Inclusion of unaffected mothers and sisters as mothers have less residual risk of developing AN	Small sample size, which may increase the likelihood of type II errors Only female participants Unaffected relatives and controls not screened for ED
Lang et al., 2016	Unaffected mothers to AN daughters screened for ED diagnostic criteria Control mothers excluded if any current/past ED or current/past ED in first- or second-degree relatives Use of valid neuropsychological measures All participants with BMI=/>30 excluded due to evidence of inefficient cognitive processing in people living with obesity	Unaffected AN mothers scored significantly higher on measures of anxiety and depression than controls, which could have affected on cognitive flexibility Only female participants Small sample size, which may increase the likelihood of type II errors
Holliday et al., 2005	Relationship between anxiety/depression scores and neuropsychological performance were analysed to control for confounding variables Discordant sibling-pair design	Small sample size, which may increase the likelihood of type II errors Only female participants
Roberts, 2009	Use of valid neuropsychological measures Unaffected sisters and control participants screened for current/past ED symptoms	Small sample size, which may increase the likelihood of type II errors Only female participants
Tenconi et al., 2010	Relatively large sample Controls with a first-degree relative with lifelong ED excluded Controls with any psychiatric illness excluded Unaffected sisters and control participants screened for current/past ED symptoms Use of valid neuropsychological measures	Unaffected sisters were not screened for other psychiatric conditions which could be confounding variables Only female participants Much smaller sample of unaffected sisters compared to AN participants and controls
Kothari, 2013	Relatively large sample Multiple births were excluded to reduce confounding variables created by different developmental trajectories Longitudinal research design Investigated maternal risk in men and women	Used phenotypes—restricting, bingeing, purging rather than AN/BN so could only include restrictive phenotypes as AN No demographic data provided for unexposed children for reaction times Research design meant that certain factors (set-shifting etc.) previously associated with AN could not be investigated No raw scores available to calculate effect size
Roberts et al., 2013	Unaffected sisters and control participants screened for current/past ED symptoms Use of valid neuropsychological measures	Small sample size, which may increase the likelihood of type II errors Only female participants
Pappaianni et al., 2023	Familial high-risk study design with asymptomatic offspring	Small sample size, which may increase the likelihood of type II errors Only female participants No raw scores available to calculate effect size
Schaumberg et al., 2020	Investigated both men and women Large sample size Longitudinal research design	Due to research design, were unable to investigate set-shifting or central coherence Not all participants completed all neuropsychological measures

Note. AN = anorexia nervosa, ED = eating disorder.

### Limitations

The strengths and limitations of the included studies are depicted in Table 3. The majority of the included studies had small sample sizes, which is a limitation as it decreases the statistical power of the findings, but is somewhat expected in this area of research as AN is rare and therefore finding large samples of participants can be difficult (van Eeden et al., 2021). The study by Kanakam et al*.* (2013) had a very small sample size, particularly in the unaffected cotwin group, perhaps explaining why they yielded different results for cognitive flexibility and central coherence compared with the other studies because, as previously discussed, the smaller sample size may have increased the risk of type II errors. Most studies only included female participants, making findings less generalizable to the population as a whole. Roberts (2009) screened unaffected sisters and controls for EDs but did not exclude unaffected sisters with anxiety and depression diagnoses. As set-shifting has been demonstrated to be a potential endophenotype for other psychiatric conditions including OCD (Muller et al., 2021) and depression (Liu et al., 2021), it is possible that the findings from this research may have reflected unrelated neuropsychological risk profiles. Some studies investigated the neuropsychological profile of children born to mothers with AN (Kothari, 2013; Pappaianni et al., 2023), but this may present a confounding variable for the findings. It is possible that the mothers having AN may have influenced the cognitive development of the children. In another study, which was not included in the current review because the presence of AN was self-reported, it was found that children born to mothers with AN had significantly lower IQ scores than those who were born to mothers who did not report a history of AN (Kothari et al., 2014). As the studies included in this review and previous studies (Schilder et al., 2017) have found the general intelligence of individuals with AN to be no different than control participants, this suggests that maternal AN may have some impact on children’s cognitive development. A further study found that children born to mothers with histories of EDs demonstrated delayed neurocognitive development and delayed head circumference growth, suggesting that maternal EDs may affect physical development as well as neuropsychological development (Koubaa et al., 2013). This was believed to be associated with prenatal malnutrition or maternal stress (Koubaa et al., 2013). Furthermore, all of the included studies were conducted in Western countries, most commonly the UK. As stated previously, there is very limited research of AN in non-Western countries (van Eeden et al., 2021), which reduces the generalizability of the findings to the population as a whole. Finally, as previously discussed, there were large variations in the findings between studies and as each study used different neuropsychological tests and methods, it is not possible to directly compare them. This moderates the conclusions that can be drawn.

There were also several limitations of the review itself. As previously discussed, the research included in the review did not investigate concordant family members with AN, therefore the findings did not meet the criteria for biomarkers to be classified as endophenotypes. Furthermore, the studies included did not investigate all of the related neuropsychological abilities. The majority investigated cognitive flexibility which provides valuable information additional to the existing research. Unfortunately, only half of the included studies investigated central coherence, which limits the information that can be gained. This was also the case for decision making abilities, as only one study investigated this, but as this study did find decision making to be impaired in first-degree relatives of individuals with AN, it may be an area for future researchers to investigate further.

### Clinical Implications

The findings from this review suggest that cognitive inflexibility and weak central coherence may be endophenotypes for AN. However, this is not robust enough due to a number of methodological limitations in the reviewed studies. More research is required to solidify these claims. Although Metacognitive training (Buttelmann and Karbach, 2017) and cognitive remediation therapy (Rhind et al., 2022; Tchanturia et al., 2007) are evidence-based interventions to improve cognitive flexibility and central coherence, these interventions will be premature to use at this stage.

### Future Research Directions

Future studies need to address current methodological limitations by recruiting larger sample sizes and using valid neuropsychological tests to assess cognitive flexibility and central coherence, such as the WCST (Faustino et al., 2021) and ROCFT (Poulton and Moffitt, 1995). Future research needs to investigate first-degree relatives of individuals with AN concordant for AN, to investigate whether reduced cognitive flexibility and impaired central coherence co-segregate with AN within families. Studies will need to study and correlate neuroanatomical and neurochemical endophenotypes that are associated with this neuropsychological endophenotype.

## CONCLUSION

In conclusion, the majority of studies included in this review suggest that reduced cognitive flexibility and weak central coherence could be endophenotypes of AN. The findings for impaired cognitive flexibility were more robust than those for weak central coherence, but this still presents as a promising endophenotype. The findings from the included studies met four out of five criteria for a biomarker to be classified as an endophenotype for both reduced cognitive flexibility and weak central coherence. More research investigating the fifth criterion, whether the biomarker co-segregates with AN within families, needs to be conducted to allow for official classification as endophenotypes of AN. This research does not suggest that there are any differences in IQ or any other neuropsychological abilities in individuals prior to the development of AN. AN can have serious negative health outcomes and be life-threatening for those diagnosed. It may be possible for screeners to be developed to identify individuals with reduced cognitive flexibility and weak central coherence who may be at risk of developing AN to allow for early identification. Perhaps interventions such as meta-cognitive training could be helpful for improving these neuropsychological abilities to potentially prevent the development of AN.

## Supplementary Material

Appendix_A_acae072

## Data Availability

All data and materials mentioned in this systematic review are publicly available. Template data collection forms can be found in the appendix of this review. Data extracted can be accessed via the relevant papers through the references provided.

## Registration and protocol

This systematic review was registered with Prospero international prospective register of systematic reviews. Registration number: CRD42023401268. The protocol can be found on the Prospero website.

## Support

This systematic review did not receive any financial support/funding.
