# Supplementary material for: A Systematic Review to Explore a Neuropsychological Profile that Predates Anorexia Nervosa
Source: Arch Clin Neuropsychol. 2024 Sep 7;40(2):330–44. doi: 10.1093/arclin/acae072 (PMC11836713; doi:10.1093/arclin/acae072)
Supplement: Appendix_A_acae072 [file appendix_a_acae072.docx]

Appendix A

**Data extraction form**

| **Review title or ID** |
| --- |
|  |

| **Study ID** *(surname of first author and year first full report of study was published e.g. Smith 2001)* |
| --- |
|  |

| **Report IDs of other reports of this study** *(e.g. duplicate publications, follow-up studies)* |
| --- |
|  |

| **Notes:** |
| --- |

**General Information**

| **Date form completed *(dd/mm/yyyy)*** | |  |
| --- | --- | --- |
| **Name/ID of person extracting data** | |  |
| **Report title**  *(title of paper/ abstract/ report that data are extracted from)* | |  |
| **Report ID**  *(if there are multiple reports of this study)* | |  |
| **Reference details** | |  |
| **Report author contact details** | |  |
| **Publication type**  *(e.g. full report, abstract, letter)* | |  |
| **Study funding source**  *(including role of funders)* | |  |
| **Possible conflicts of interest**  *(for study authors)* | |  |
| **Notes:** |  | |

**Eligibility**

| **Study Characteristics** | | **Review Inclusion Criteria**  *(Insert inclusion criteria for each characteristic as defined in the Protocol)* | **Yes/ No / Unclear** | **Location in text**  *(pg & ¶/fig/table)* |
| --- | --- | --- | --- | --- |
| **Type of study** | | Randomised trial |  |  |
|  |  | Non-randomised trial |  |  |
|  |  | Controlled before-after study   - Contemporaneous data collection - At least 2 intervention and 2 control clusters |  |  |
|  |  | Interrupted time series OR  Repeated measures study   - At least 3 timepoints before and 3 after the intervention - Clearly defined intervention point |  |  |
|  |  | Other design (specify): |  |  |
| **Participants** | |  |  |  |
| **Types of intervention** | |  |  |  |
| **Types of outcome measures** | |  |  |  |
| **Decision:** | |  | | |
| **Reason for exclusion** | |  | | |
| **Notes:** |  | | | |

**DO NOT PROCEED IF STUDY EXCLUDED FROM REVIEW**

**Population and setting**

|  | | **Description**  *Include comparative information for each group (i.e. intervention and controls) if available* | **Location in text**  *(pg & ¶/fig/table)* |
| --- | --- | --- | --- |
| **Population description**  *(from which study participants are drawn)* | |  |  |
| **Setting**  *(including location and social context)* | |  |  |
| **Inclusion criteria** | |  |  |
| **Exclusion criteria** | |  |  |
| **Method/s of recruitment of participants** | |  |  |
| **Notes:** |  | | |

**Methods**

|  | | **Descriptions as stated in report/paper** | **Location in text**  *(pg & ¶/fig/table)* |
| --- | --- | --- | --- |
| **Aim of study** | |  |  |
| **Design**  *(e.g. parallel, crossover, non-RCT)* | |  |  |
| **Start date** | |  |  |
| **End date** | |  |  |
| **Duration of participation**  *(from recruitment to last follow-up)* | |  |  |
| **Notes:** |  | | |

**Risk of Bias assessment**

*See* [*Chapter 8*](http://www.mrc-bsu.cam.ac.uk/cochrane/handbook/index.htm#chapter_8/8_assessing_risk_of_bias_in_included_studies.htm) *of the Cochrane Handbook. Additional domains may be required for non-randomised studies.*

| **Domain** | **Risk of bias**  *Low/ High/Unclear* | **Support for judgement** | **Location in text**  *(pg & ¶/fig/table)* |
| --- | --- | --- | --- |
| **CASP** |  |  |  |

**Participants**

*Provide overall data and, if available, comparative data for each intervention or comparison group.*

|  | | **Description as stated in report/paper** | **Location in text**  (pg & ¶/fig/table) |
| --- | --- | --- | --- |
| **Total no. randomised**  *(or total pop. at start of study for NRCTs)* | |  |  |
| **Clusters**  *(if applicable, no., type, no. people per cluster)* | |  |  |
| **Baseline imbalances** | |  |  |
| **Withdrawals and exclusions**  *(if not provided below by outcome)* | |  |  |
| **Age** | |  |  |
| **Sex** | |  |  |
| **Race/Ethnicity** | |  |  |
| **Severity of illness** | |  |  |
| **Co-morbidities** | |  |  |
| **Other treatment received**  *(additional to study intervention)* | |  |  |
| **Other relevant sociodemographics** | |  |  |
| **Subgroups measured** | |  |  |
| **Subgroups reported** | |  |  |
| **Notes:** |  | | |

**Outcomes**

*Copy and paste table for each outcome.*

**Outcome 1**

|  | | **Description as stated in report/paper** | | **Location in text**  *(pg & ¶/fig/table)* |
| --- | --- | --- | --- | --- |
| **Outcome name** | |  | |  |
| **Time points measured**  *(specify whether from start or end of intervention)* | |  | |  |
| **Time points reported** | |  | |  |
| **Outcome definition**  *(with diagnostic criteria if relevant and note whether the outcome is desirable or undesirable if this is not obvious)* | |  | |  |
| **Person measuring/ reporting** | |  | |  |
| **Unit of measurement**  *(if relevant)* | |  | |  |
| **Scales: upper and lower limits**  *(indicate whether high or low score is good)* | |  | |  |
| **Is outcome/tool validated?** | | *Yes/No/Unclear* |  |  |
| **Imputation of missing data**  *(e.g. assumptions made for ITT analysis)* | |  | |  |
|  | |  | |  |
| **Notes:** |  | | | |

**Outcome 2**

|  | | **Description as stated in report/paper** | | **Location in text**  *(pg & ¶/fig/table)* |
| --- | --- | --- | --- | --- |
| **Outcome name** | |  | |  |
| **Time points measured**  *(specify whether from start or end of intervention)* | |  | |  |
| **Time points reported** | |  | |  |
| **Outcome definition**  *(with diagnostic criteria if relevant and note whether the outcome is desirable or undesirable if this is not obvious)* | |  | |  |
| **Person measuring/ reporting** | |  | |  |
| **Unit of measurement**  *(if relevant)* | |  | |  |
| **Scales: upper and lower limits**  *(indicate whether high or low score is good)* | |  | |  |
| **Is outcome/tool validated?** | | *Yes/No/Unclear* |  |  |
| **Imputation of missing data**  *(e.g. assumptions made for ITT analysis)* | |  | |  |
|  | |  | |  |
| **Notes:** |  | | | |

**Applicability**

| **Have important populations been excluded from the study?**  *(consider disadvantaged populations, and possible differences in the intervention effect)* | | *Yes/No/Unclear* |  |  |
| --- | --- | --- | --- | --- |
| **Is the intervention likely to be aimed at disadvantaged groups?**  *(e.g. lower socioeconomic groups)* | | *Yes/No/Unclear* |  |  |
| **Does the study directly address the review question?**  *(any issues of partial or indirect applicability)* | | *Yes/No/Unclear* |  |  |
| **Notes:** |  | | | |

**Other information**

|  | | **Description as stated in report/paper** | **Location in text**  *(pg & ¶/fig/table)* |  |
| --- | --- | --- | --- | --- |
| **Key conclusions of study authors** | |  |  |  |
| **References to other relevant studies** | |  |  |  |
| **Correspondence required for further study information**  *(what and from whom)* | |  | |  |
| **Further study information requested**  *(from whom, what and when)* | |  | |  |
| **Correspondence received**  *(from whom, what and when)* | |  | |  |
| **Notes:** |  | | | |
